# Evaluation of Knowledge, Perception, and Risk Awareness About Breast Cancer and Its Treatment Outcome Among University of Gondar Students, Northwest Ethiopia

**DOI:** 10.3389/fonc.2018.00501

**Published:** 2018-11-02

**Authors:** Begashaw Melaku Gebresillassie, Eyob Alemayehu Gebreyohannes, Sewunet Admasu Belachew, Yohannes Kelifa Emiru

**Affiliations:** School of Pharmacy, College of Medicine and Health Science, University of Gondar, Gondar, Ethiopia

**Keywords:** breast cancer, awareness, self-examination of breast, treatment-outcome, Ethiopia

## Abstract

**Background:** Breast cancer is among the most common life-threatening public health problems of global concern including Ethiopia. Knowledge and awareness about the disease will help to reduce the number of cases who present at late stages of the disease. The objective of this survey was to assess the knowledge, perception and risk awareness about breast cancer among female medical and health science students of University of Gondar, Ethiopia.

**Methods:** A cross sectional survey was conducted from May 03 to June 01, 2017 at University of Gondar, Ethiopia. Three hundred students were proportionally selected from nine departments using simple random sampling method. Using a structured questionnaire data on risk factors, symptoms and perception about breast cancer and its management approach was collected. Data were entered to and analyzed using SPSS version 21.

**Results:** A total of 300 students had fully completed the survey making the response rate 95.24. The participants' mean age was 21.4 years with the standard deviation (SD) of 2.13 years. The overall level of knowledge on breast cancer was low. Majority of the participants were unaware for complex risk factors such as first child after the age of 30 years (51%), early onset of menses (55.3%), and menopause after the age of 55 years (47.7%) are liked with breast cancer even though they acknowledged old age, family history, and smoking as possible risk factors for breast cancer. Pain in the breast region, change in the shape of the breast, and nipple discharge were the most frequently correctly identified symptoms of breast cancer. Majority of the study participants had also correct beliefs about breast cancer management and its outcomes. however, they had negative perception of breast cancer treatment by considering it to be a long-term and painful process. In binary logistic regression analysis department (*p* = 0.000) and year of study (*p* = 0.008) were found to be an independent predicting factors for knowledge among the study participants.

**Conclusions:** The overall level of knowledge on breast cancer and clinical breast examination guidelines was found to be low even though majority had positive perception toward the treatment and its outcomes. Hence, intensive breast health awareness campaign, which should also stress on the importance of early detection and reporting, is necessary to improve the knowledge about breast cancer.

## Introduction

Breast cancer is one of the top most public health concern jeopardizing the lives of many peoples worldwide (1, 2). This kind of cancer is malignant by nature endangers breast tissue, and may involve either the tubules carrying milk or ducts which produce the milk. This type of disease can metastasize to distant areas or invade surrounding tissues. Commonly, the disease happens in women population although males may also suffer from it (3).

Globally, the incidence of breast cancer has increased from 641 000 in 1980 to 1 643 000 in 2010 with an annual increment of 3.1% (4). Different reports revealed that in sub-Saharan African and other resource limited countries the rate of occurrence of breast carcinoma is significantly increasing (5, 6). The overall incidence in Ethiopia is also increasing, it is estimated around 10,000 women and men taking in consideration that more number of cases were unreported since women from country side usually prefer to go to cultural treatment providers than looking for health care institution (7). Some of contributing factors implicated in steady rise in breast cancer incidence in developing countries are widespread urbanization, changing patterns of reproductive and environmental risks factors, obesity, decreased physical activity, and increasing life expectancy (8, 9). Since 1980, the mortality of breast cancer has also increased from 250,000 to 425,000 in 2010. About 60% of deaths from breast cancer are occurring in low income countries (4).The high mortality associated with breast cancer in countries like Ethiopia is most importantly due to knowledge shortage about the disease that may leads to late diagnosis (10).

The exact reasons for the occurrence of breast cancer have not been fully understood. However, numerous researchers have identified a number of predictors that can increase one's likelihood of getting breast cancer. They called them risk factors and includes family and individual backgrounds of breast cancer; delayed menopause (>55 years); early menarche (<12 years); late age at first full-term pregnancy (>30 years); alcohol use; aging; never breastfeeding a child; exposure to contraceptives; tobacco smoking; high fat diet; obesity (postmenopausal); recent and long-term consumption of hormonal replacements; physical inactivity; high-dose x-ray to chest (11, 12).

Although in developed countries, screening of such malignant tumor is usually performed using mammograms, the access to most women in sub Saharan countries are limited. From the existing situation it is uncertain to have amendments in the upcoming days (13). Breast self-examination (BSE) is the mere, reasonable and practical option of screening for women living in Africa in case of non-appearance of mammography (13, 14). With applicable training of BSE along with clinical breast examination and comprehensive health education of the disease, it is likely to early detect the disease. Women who repeatedly do BSE tend to accustom both the feeling and appearance of their breasts this in turn will help them to early identify any changes. However, if it is done improperly, there might be untrue positive or negative findings associated with poor BSE and this may raise a great disappointment to undergo mammographic screening even in the set up where it is available and easily accessible (15).

In Ethiopia poor awareness is the core concern and there is also stigma and misconceptions/understanding about cancer, that all cancer cases are incurable. Associated with this little work was done so far to promote the awareness that most cancer cases can be prevented, even cured if detected early, and quality of life can also be improved (16).

As the principal focus of the present study was students, it strengthens cancer detection and prevention strategies at an early age, creates education opportunities for shaping health behaviors into adulthood and also encourage discussions between students and their guardians as well as relatives. Therefore, the purpose of the present survey was to explore the knowledge, perception and risk awareness toward breast cancer among female medical and health science students of University of Gondar, Ethiopia.

## Methods

### Study design and setting

An institutional based cross sectional study was employed among female medical and health science students of University of Gondar (UOG). UOG is among the first born universities in Ethiopia and located 737 km from the capital of Ethiopia, in northwest direction. It has 5 campuses such as Atse Tewodros, Maraki, College of Medicine and Health Sciences (CMHS), Atse Fasil, and Meles Zenawi. At present, with the nine academic offices it offers about 56 undergraduate and 64 postgraduate programs in regular, distance, extension and summer programs.

### Study samples

Female students who had interest to complete the self-administered questionnaire were included. However, students with already existing disease and unable to understand the questionnaire were excluded.

### Sample size determination and sampling technique

Appropriate sample size was estimated using a single proportion formula (17)

$$\begin{array}{l} \text{n}={\text{z}}^{2}\text{p}\left(1-\text{p}\right)/{\text{d}}^{2} \end{array}$$

Where n is the need sample size; *d*, marginal error (*d* = 0.05); *Z*, the required degree of accuracy at 95% confidence level, which is 1.96; *P* = 0.5 (50%) level of knowledge, as there was no study conducted in the study area to the best of literature search made. Using the above formula, the sample size was calculated as follows:

$$\begin{array}{l} \text{ni }=\;{\left(1.96\right)}^{2}\left(0.5\right)\left(1-0.5\right)/{\left(0.05\right)}^{2}\\ \;=\;\left(3.8416\right)\left(0.25\right)/0.0025\\ \;=\;0.9604/0.0025\\ =385 \end{array}$$

Since the sample was drawn from the gross population of 1463, which is <10,000, the required final sample size was determined after applying the correction factor;

$$\begin{array}{l} \text{nf }=\;\frac{ni}{1+\frac{ni}{N}}\\ \text{nf }=\;385/1+385/1360\\ \;=\;385/1.283\\ \;=\;300 \end{array}$$

where

$$\begin{array}{l} nf\;=\;\text{calculated final sample size}\\ ni\;=\;\text{reduced sample size},\\ N\;=\text{ totalnumberofthesourcepopulation}. \end{array}$$

Considering 5% for probable non-respondent, the required final sample size was 315.

### Sampling technique/procedure

**Figure 1:**
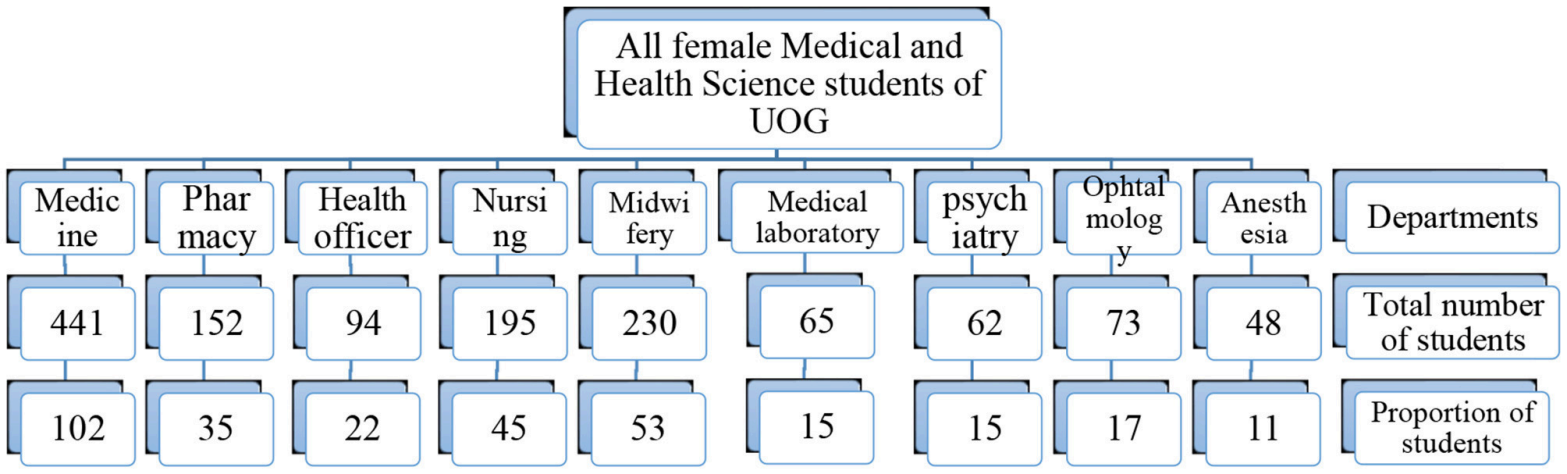
A flow chart describing the sampling technique, UOG, 2017.

The final sample was distributed proportionally across different departments. Using simple random sampling technique the required samples from each department were recruited in the study (Figure 1).

### Data collection technique and management

Three principal investigators were responsible for conducting the data collection. The data were obtained through self-administered questionnaire. The tool used was adopted from prior studies conducted in this area and prepared in English (18–23). The data collection tool has three sections: The first part includes socio-demographic variables while the second one comprises knowledge of breast cancer risk factors, symptoms and screening tests and the third part focuses on perception of management and outcomes. Participants' response was given 1 point for correct and zero for incorrect answer or do not know. The knowledge status was considered as “low,” “moderate,” and “high” if the scores fall in the range of 0–49, 50–79, and 80–100%, respectively (24). Likert scale of 1–5 was also employed to rate the responses of participants regarding perception related questions. It was also pretested on 25 participants who were not included in the final analysis before the commencement of actual data collection. Moreover, training on the familiarization of the instrument and strategies to approach the students were provided for the investigators.

### Data entry and analysis

The collected data using quantitative method was entered to and analyzed using Statistical packages for social sciences (SPSS) version 21 statistical software. Frequencies, percentages, tables, flow chart were used to describe study variables. Binary logistic regression analysis was done to examine the association among different variables. For determining statistically significant associations, *P* < 0.05 and confidence interval (CI) of 95% were used as cut points.

### Ethical considerations

The study was ethically approved by the ethical review committee of School of Pharmacy (SoP), University of Gondar (UoG) with an approval number of UoG-SoP-140/2017. Informed consent was gained from each participant before the start of the study. They were also informed that involvement in the study was fully voluntarily and withdrawal at any stage if they refused to participate was assured. Information obtained from the survey was kept confidential. In addition, patient identifiers were not used.

## Results

### Socio-demographic characteristics of the participants

In the current study, out of the total interview guides/questionnaires of sample of 315 students who were interviewed 300 were included in the analysis, and 15 encounters were excluded due to incompleteness making the response rate 95.24%. The average age of the study respondents were 21.4 years with the standard deviation (SD) of 2.13 years.

More than two third 188 (62.7%) of the participants had no previous participation in breast care awareness. However, they had interest to participate in activities that encourages breast cancer awareness 234 (78.0%) (Table 1).

**Table 1:** Distribution of participants by socio-demographic characteristics, UOG, 2017.

**Variable**		**Frequency**	**Percent**
Age (in years)	18–25	291	97
	>25	9	3
Previous participation in breast care awareness	Yes	112	37.3
	No	188	62.7
Interest in activities to promote breast awareness	Yes	234	78.0
	No	66	22
Department	Medicine	97	32.3
	Pharmacy	33	11.0
	Midwifery	51	17.0
	Nurse	43	14.3
	Psychiatry	14	4.6
	Health officer	21	7.0
	Medical laboratory	15	5.0
	Optometry	16	5.3
	Anesthesia	10	3.3
Year of study	First year	36	12
	Second year	108	28.3
	Third year	68	22.7
	Fourth year	93	31.0
	Fifth year	18	6.0
Monthly income (in ETB)	<1,000	194	64.7
	>= 1,000	106	35.3

### Knowledge of breast cancer symptoms, risk factors, and screening test

More than two third of the participants acknowledged old age, family history, and smoking as possible risk factors for breast cancer. Majority of the participants were unaware for complex risk factors such as first child after the age of 30 years (51%), early onset of menses (55.3%), and menopause after the age of 55 years (47.7%) are liked with breast cancer. Pain in the breast region, Change in the morphology/shape of the breast and nipple discharge were the most frequently correctly identified symptoms of breast cancer. Moreover, 168 (56.0%) of the participants were aware about once a month recommendations for practicing breast self-examination (BSE) and 108 (36%) for once a year clinical breast examination (CBE) (Table 2).

**Table 2:** Participants' response to important knowledge related questions, UOG, 2017.

**Variables**	**Correct *N* (%)**	**Incorrect *N* (%)**
**GENERAL KNOWLEDGE**		
Only females are affected by breast cancer	177 (59.0%)	123 (41%)
Breast cancer can be transmitted from one person to another	242 (80.7%)	58 (19.3%)
Breast cancer is the leading cause of death in Ethiopian women	126 (42%)	174 (58%)
**KNOWLEDGE OF BREAST CANCER RISK FACTORS**		
Old age	204 (68.0)	96 (32%)
Family history of breast cancer	226 (75.3%)	74 (24.7%)
Cigarette smoking	213 (71.0%)	87 (29.0%)
Low fat diet	84 (28.0%)	216 (72.0)
First child after the age of 30 years	147 (49.0%)	153 (51%)
Early onset of menses (before the age of 12 years)	134 (44.7%)	114 (55.3%)
Late menopause (after the age of 55 years)	157 (52.3%)	143 (47.7%)
Use of oral contraceptives	182 (60.7%)	118 (39.3%)
Large breasts	225 (75.0%)	75 (25.0%)
Breast feeding	239 (79.7)	61 (20.3%)
**KNOWLEDGE OF BREAST CANCER SYMPTOMS**		
Painless breast lump	161 (53.7)	139 (46.3%)
Lump under armpit	171 (57%)	129 (43%)
Nipple discharge	205 (68.3)	95 (31.7%)
Change in the shape of the breast	223 (74.3%)	77 (25.7)
Pain in the breast region	234 (78%)	66 (22%)
Dimpling of breast	195 (65%)	105 (35%)

The overall level of knowledge was described by nineteen questions on breast cancer for general knowledge, risk factors and symptoms of breast cancer. Although the overall level of knowledge on breast cancer was low, high level of knowledge was observed in questions related to general knowledge about breast cancer 70 (23.3%) (Table 3).

**Table 3:** Knowledge about breast cancer, UOG, 2017.

**Variables**	**Level of knowledge**			**Mean ± SD**
	**Low frequency (%)**	**Moderate frequency (%)**	**High frequency (%)**	
General knowledge	103 (34.3)	127 (42.3)	70 (23.3)	1.82 ± 0.88
Knowledge of breast cancer risk factors	123 (41.0)	134 (44.7)	43 (14.3)	5.06 ± 2.71
Knowledge of breast cancer symptoms	195 (65.0)	73 (24.3)	32 (10.7)	2.02 ± 1.62
Overall knowledge on breast cancer	185 (61.7)	103 (34.3)	12 (4.0)	4.81 ± 5.13

### Perception toward breast cancer treatment and its outcomes

Majority of study participants had correct beliefs about breast cancer management and its outcomes. however, they had negative perception of breast cancer treatment by considering it to be a long-term and painful process (Table 4).

**Table 4:** Perception toward breast cancer treatment and its outcomes, UOG, 2017.

**Items**	**SA**	**A**	**N**	**D**	**SD**
A woman after receiving treatment for breast cancer can enjoy a good quality of life.	90 (30%)	140 (46.7%)	30 (10.0%)	37 (12.3%)	3 (1%)
The treatment for breast cancer is a long and painful process.	64 (21.3%)	127 (42.3%)	65 (21.7%)	35 (11.7%)	9 (3%)
Treatments for breast cancer are more helpful to young people.	91 (30.3%)	136 (45.3%)	46 (15.3%)	22 (7.3%)	5 (1.7%)
Treatment for breast cancer is embarrassing.	31 (10.3%)	58 (19.3%)	81 (27%)	71 (23.7%)	59 (19.7%)
Treatment of breast cancer results in loss of physical beauty	66 (22%)	109 (36.3%)	47 (15.7%)	49 (16.3%)	29 (9.7%)

*SA, Strongly agree; A, Agree; N, Neutral; DA, Disagree; SDA, Strongly disagree*.

### Predictors of knowledge of breast cancer among the participants

In binary logistic regression analysis department and year of study were found to be an independent predicting factors for knowledge among the participants. Students in pharmacy department (AOR = 0.839, CI = 0.312–2.255) were found to have 16.1% less likely to have good knowledge of breast cancer compared to public health officer students. On the other hand, students under first year of study (AOR = 2.661, CI = 0.407–17.389) were found to have 1.661 times more likely to have poor knowledge of breast cancer compared to fifth year students (Table 5).

**Table 5:** Predictors of knowledge of breast cancer among the participants, UOG, 2017.

**Variables**	**Knowledge**		**AOR (95% CI)**	***P*-value**
	**Good**	**Poor**		
**Age (in years)**				0.644
18–25	213	78	0.688 (0.141–3.356)	
>25	6	3	1	
**Previous participation in breast care awareness**				0.156
Yes	89	23	0.635 (0.339–1.189)	
No	130	58		
**Department**				0.000^*^
Medicine	82	15	8.218 (2.96–22.816)	
Pharmacy	17	16	0.839 (0.312–2.255)	
Midwifery	42	9	2.154 (0.815–5.693)	
Nursing	31	12	12.553 (1.296–47.805)	
Medical laboratory	5	10	3.127 (0.628–15.573)	
Anesthesia	7	3	5.967 (1.170–30.424)	
Psychiatry	5	9	2.209 (0.573–8.518)	
Ophthalmology	12	4	0.692 (0.173–2.767)	
Public health officer	18	3	1	
**Year of study**				0.008^*^
First year	19	17	2.661 (0.407–17.389)	
Second year	59	26	2.329 (0.442–12.276)	
Third year	48	20	2.831 (0.549–14.599)	
Fourth year	77	16	0.625 (0.112–3.481)	
Fifth year	16	2	1	
**Monthly income (in ETB)**				0.653
<1,000	141	53	1.159 (0.609–2.206)	
>= 1,000	78	28	1	

* *Statistically significant*.

## Discussion

It is an unconcealed evidence that breast cancer turn out to be one of the frequently occurring cancers among female population of Ethiopia and barriers associated with detection and management of the case decreases survival rates (1, 25). The present study indicates that huge number of the study respondents had no previous involvement in breast care awareness. However, they had interest to involve in activities to encourage breast cancer awareness. This finding concurs with the report from United Arab Emirates (19).

This study stated that, beyond two third of the study subjects revealed to have low overall knowledge toward risk factors, general knowledge, and clinical manifestations of the malignant tumor, which was similar with the reports from Malaysia, Saudi, Egypt, and Nigeria (21, 26–28). Many female students in such higher institutions are unacquainted of clinical breast examination (CBE), the predictors, and early presentation of the tumor. This might be the cause for the delayed presentation of the disease in developing countries. Therefore, it is crucial to provide comprehensive breast cancer and health awareness programs for female youngsters.

Bulk of the respondents acknowledged old age, family history, and tobacco use as potential predisposing factors for the tumor. Most of the participants were also unaware of complex risk factors such as first child after the age of 30 years (51%), early onset of menses (55.3%), and menopause after the age of 55 years (47.7%) are liked with breast cancer. This finding was comparable with studies done in Malaysia, Egypt, Oman and Britain (18, 22, 23, 29).

Most of the study participants identified pain in the breast region as a symptom for breast cancer followed by swing in the morphology/shape of the breast and nipple discharge. However, more than one third of the respondents had less knowledge about under armpit lump and painless breast lump as cautioning signs of the disease. This finding concurs with the reports from Malaysia, Egypt, Nigeria, and Oman (18, 22, 23, 28). However, it was higher when matched to the findings from Madawolabo (30). This could be justified as the respondents were merely from medical and health science streams, it was expected that awareness about manifestations of the disease to be good when matched to other technology campuses in the university.

Numerous respondents had the factual perception about breast cancer management and its outcomes. however, they had negative view of the tumor treatment by seeing the length of treatment and painful process, which was similar with the report from Malaysia and Nigeria (18, 28). This finding suggest the need for customized educational interventions using different lines such as social media, distribution of leaflets, television/ radio broadcasts and proper counseling as tools for improving the knowledge and perception about the tumor and the treatment outcomes.

Binary logistic regression analysis was performed and factors such as department and study year were recognized as independent predictors for disease related knowledge among the study participants. Pharmacy students (AOR = 0.839 CI = 0.312–2.255) were 16.1% less likely to own good knowledge of breast cancer when equated to public health officer students. The study also mentioned that the odds of having poor knowledge toward the disease were 1.661 times higher among students enrolled under first year of study (AOR = 2.661, CI = 0.407–17.389) compared to fifth year students. This might be due more exposure and familiarization about breast cancer and related health issues in different courses, trainings and seminars will be embraced as academic year goes up.

As a drawback, although this research is the first in its kind in the study setting that can be used as an input for implementing basic projects, it is conducted only in one institution that the generalization to all over university students will be in question. With this, we highly recommended a large scale and multi centered survey that comprises diverse participants to validate our output and provide more representative findings.

## Conclusions

In this study, the overall level of knowledge on breast cancer was low. Majority of the participants were unaware for complex risk factors such as first child after the age of 30 years, early onset of menses, and menopause after the age of 55 years are liked with breast cancer. However, small proportion of the participants were aware about clinical breast examination guidelines. Pain in the breast region, change in the shape of breast and nipple discharge were the most frequently correctly identified symptoms of breast cancer. On the other hand, majority of the participants acknowledged old age, family history, and smoking as possible risk factors for breast cancer and they had also positive perception toward breast cancer treatment and its outcomes. Department and year of study were found to be an independent predicting factors for knowledge among the participants.

## Implications

Bearing in mind the greatest prominence of knowledge, perception and risk awareness about such tumor, institutions and different stakeholders working on cancer should offer a tailored health promotion and awareness creation to university youngsters, in line with cultivating facilities that let them do the screening examination regularly. In addition, the country ministry of education and health has to work on incorporating capacity building regular trainings regarding such disease avoidance and early detection in such organizations.

## Author contributions

All authors listed have made a substantial, direct and intellectual contribution to the work, and approved it for publication.

### Conflict of interest statement

The authors declare that the research was conducted in the absence of any commercial or financial relationships that could be construed as a potential conflict of interest.
